# Contrasting alien effects on native diversity along biotic and abiotic gradients in an arid protected area

**DOI:** 10.1038/s41598-021-92763-2

**Published:** 2021-06-30

**Authors:** Reham F. El-Barougy, Ibrahim A. Elgamal, Abdel-Hamid A. Khedr, Louis-Félix Bersier

**Affiliations:** 1 Department of Botany and Microbiology, Faculty of Science, Damietta University, New Damietta, Egypt; 2 Department of Biology, Ecology and Evolution unit, University of Fribourg, Chemin du Musée 10, 1700 Fribourg, Switzerland; 3 Nature Conservation Sector, Egyptian Environmental Affairs Agency, Cairo, 11728 Egypt

**Keywords:** Community ecology, Ecological modelling, Plant ecology, Phylogenetics

## Abstract

Alien impact on native diversity could be a function of both the relatedness of alien species to native community and resources availability. Here, we investigated whether alien plants expand or decrease the functional and phylogenetic space of native plant communities, and how this is affected by alien relatedness to natives and by resources availability. We used a trait-environment dataset of 33 alien and 130 native plants in 83 pairs of invaded and non-invaded plots, covering a gradient of soil resources (organic matter-nitrogen) in Saint-Katherine-Protectorate, Egypt. First, we compared the changes in native composition and calculated alien relatedness to natives within each pair of plots. Second, we tested the effects of resources availability and relatedness on the magnitude of alien impact (defined as a change in native diversity). We found that native composition was phylogenetically less but functionally more diverse in invaded plots compared to non-invaded ones. Moreover, in resources-rich plots, dissimilar aliens to natives significantly increased native diversity, while in resource-limited ones, similar aliens to natives declined native diversity. These results suggest that the assessment of alien impacts in arid-regions is significantly linked to resources-availability and relatedness to natives. Hence, future studies should test the generality of our findings in different environments.

## Introduction

Invasions by alien plant species are increasingly impacting native biodiversity, communities and ecosystem functioning^1–3^. Invasive alien plants can cause a local loss of native species richness and change the dominance relationships in a community, and consequently alter ecosystem processes^1,4–7^. The awareness of these potential consequences of biological invasions on native biodiversity is increasing. However, still little is known about how biological invasions affect taxonomic, phylogenetic and functional aspects of biodiversity. Furthermore, how the impacts depend on the functional and phylogenetic relatedness (similarity or dissimilarity of alien species to native species), and how the impacts vary along gradients of abiotic environmental conditions deserves more research.

Alien species are likely to change the richness, functional and phylogenetic diversity of the native resident community. The direction of this change could depend on the ecological similarity or dissimilarity between aliens and natives, and on resources availability^8–11^. In this sense, Darwin's naturalization conundrum (DNC; Darwin 1859), which includes both pre-adaptation and naturalization as important factors for alien success, has encountered a considerable amount of attention^12–15^. According to the pre-adaptation hypothesis, if an alien species is phylogenetically closely related to native resident species, it will likely share similar traits and occupy a similar niche as the closely-related species due to the strong effect of environmental filtering^12,16^. This filtering restricts the range of trait values of the alien species to be more similar to those of natives^17–23^. For example, in resource-limited environments, a pattern of aliens co-occurring with functionally similar natives is expected. This increasing similarity might cause the alien species to replace natives and to occupy a portion of the functional and possibly of the phylogenetic space originally occupied by the native species they replaced, leading to the contraction of the functional and phylogenetic diversity of the native resident communities^24,25^.

According to Darwin’s Naturalization hypothesis (DNH), if the alien species is distantly related to resident natives, it will occupy a distinct niche and its competitive effect is assumed to be low with resident natives. Under this scenario, alien species can fill vacant niches that are not used by natives leading to niche differentiation. For example, in resource-rich environments, exploitative aliens might be more likely to naturalize if they are phylogenetically and functionally dissimilar to the native species, because then they can exploit unfilled ecological niches^26^. Such alien species could expand the native phylogenetic and functional diversity, compared to non-invaded communities^27^, and provide a novel suit of traits and evolutionary origins to the invaded communities^28–30^.

Significantly, competitive interactions between aliens and natives within low and high resource environments can also cause the dominance of clades of native species bearing traits related to greater competitive abilities^31,32^. This dominance arises from the relative differences in the competitive abilities among alien and native species^11,31,33–36^. Such competition can select strong competitors among the environmentally adapted aliens that can coexist within local native communities, leading to a decline in native richness and abundance^37–42^. For example, natives are expected to be eliminated by aliens if natives are weak competitors and overlap too much in their soil preferences with aliens, and vice-versa^28,31^. This phenomenon is linked to the tendency of alien species to become dominant in a plant community outside its native range and to locally replace native species^37–39^.

In this study, we investigated (1) whether the presence of alien plants changes the richness and abundance of natives, (2) whether alien plants occupy a portion of the functional and phylogenetic space of native communities, (3) whether alien plants can expand the trait space and phylogenetic (evolutionary) space to the native community, (4) to what extent the impact of alien plants on native communities is affected by soil resources availability and (5) by alien relatedness to natives.

We expect an increase in the richness, functional and phylogenetic space of invaded-native communities compared to non-invaded ones in resources rich environments. This increase could be attributed to the presence of alien plants that tend to be functionally and phylogenetically dissimilar to natives (i.e. Darwin’s Naturalization Hypothesis). This dissimilarity enables alien plants to employ different strategies of resource use (i.e. niche differentiation and fitness differences), exploit resources unused by natives, and fill niches unoccupied by natives. As a consequence of dissimilarity, a competitive exclusion^10,31,43,44^ may arise to eliminate alien /native species that are too similar in their trait values^45–47^.

By contrast, in limited resources environments, we expect a decline in the native diversity measures (richness, functional and phylogenetic matrices) as well as the phylogenetic signal of native traits in invaded communities compared to non-invaded ones. This could be attributed to the negative impact of alien plants on resident natives if aliens and natives are functionally and phylogenetically similar (i.e. Darwin’s Pre-adaptation Hypothesis). In this case, aliens and natives have similar fitness and equivalent competitive effects or sharing similar niches due to the effect of environmental filtering that will cause alien plants to occupy a portion of the phylogenetic and functional space originally occupied by natives.

## Methods

### Vegetation plots, species inventories and soil data

Field surveys were carried out during the spring and summer seasons (March to July) of 2018 in Saint Katherine Protectorate (SKP), South Sinai, Egypt (Supplementary Appendix A). The diversity in geomorphological and geological structures of SKP resulted in six types of microhabitats, namely Wadis (valleys), Terraces, Slopes, Gorges, Cliffs, Farsh (basins) and Caves (SKP Management Plan 2003^48,49^). Wadis are the most common microhabitats in the current study area, and act as drainage systems collecting water from catchment areas for plant growth. The wadis in SKP are very narrow, have very steep slopes, short in length and occur at higher elevations, ranging from 1190 to 1900 m (a.s.l). Across the entire study area, we selected 83 plots of 10 m^2^ that were invaded by at least one alien plant species. The maximum number of alien species in a plot was three. Close to each invaded plot, at approximately 10 m distance, we set up a 10 m^2^ plot with similar vegetation but with no invader. This resulted in 83 pairs of invaded and non-invaded plots. We identified all plants to species (33 aliens and 130 natives), and we counted the numbers of alien and native individuals per plot (Supplementary Appendix A, Supplementary Table A1).

To describe the abiotic environment and resource availability, we obtained for each plot measurements of soil moisture, soil nitrogen, and organic matter content. Soil moisture measurements were taken directly in the field in the early morning, using soil hygrometer. Three soil samples were collected at randomly chosen positions within each plot and air-dried to constant mass. Then, soil water extracts at 1:5 were prepared for the determination of soil nitrogen and organic matter content. The percentage of organic matter was calculated as the difference between total C and CaCO_3_%^50^. The total concentration of available nitrogen (mg/L) was calculated using a CHN analyser (EA1108, Carlo Erba Instruments, USA) and standard methods^51^. These measures were highly correlated; we created a compound variable, named “soil resources”, as the coordinates of the first axis of a PCA for these three variables (the first axis accounted for 90% of the total variability).

### Species trait data

To quantify the functional similarity of the 33 alien and 130 native species, we measured three traits non-destructively on all plants in the plots. These traits were plant height from the ground [cm], the number of leaves, and the number of reproductive organs (flowers and fruits). In addition to these traits, we also determined specific leaf area (SLA [cm^2^/g]) and aboveground biomass [kg]. For the alien plants, these two traits were measured destructively on individuals collected from the field plots. For each plant, we scanned the leaves and measured the total leaf area using the IMAGEJ software, version 1.49^52^. Then, we determined the leaf dry weight, and calculated the SLA as the leaf area divided by the leaf weight^53^. To obtain measurements for above-ground biomass of alien plants, all aboveground parts (leaves and stems) were dried in a drying oven (VWR International) at 50 °C for three days, and then weighed using a Mettler Toledo ML Series Precision Balance (ML Analytical balance).

### Phylogenetic and functional trait analyses

To quantify phylogenetic diversity and relatedness, we constructed a phylogeny of the 166 species (33 aliens, 130 natives) using four commonly sequenced genes available in GenBank^54^: rbcL, matK, ITS1 and 5.8 s. Of the 133 native species, 120 species had at least one gene represented in GenBank. For the 13 native species without sequence data, we used instead the available sequences from congeneric relatives as a proxy (see phylogenetic guidelines by Jin and Cadotte 2015). We also included the genetic sequences of *Amborella trichopoda* Baill., a species that diverged early in angiosperm evolution, to serve as an outgroup species. Sequences were aligned for each of the four genes independently using FASconCAT v1.0^55^ and combined into a single matrix. We then selected best-fit maximum likelihood (ML) models of nucleotide substitution for each gene sequence by jModeltest^56^. The ML phylogeny was generated using the PhyML algorithm with a BIONJ starting tree^57,58^ to estimate the phylogeny. Nodal support was estimated using approximate likelihood-ratio test (aLRT) scores, which have been shown to correlate with ML-bootstrap scores but require much less computational time^58^. We then used a semiparametric rate-smoothing method^59^ to transform the phylogeny to an ultrametric tree using the R package ape^60^.We iterated these functions across a suite of rate-smoothing parameters and found that the parameter value that maximized the likelihood was ʎ = 1. The final ultrametric phylogenetic tree, including 130 native species and 33 alien species, is provided in Supplementary Appendix A, Supplementary Fig. A.

We calculated Faith’s phylogenetic diversity (PD), which quantifies the total independent evolutionary history of a subset of taxa^61,62^ of all native species in each of the 166 plots. We also calculated the native phylogenetic relatedness (NMPD) within each non-invaded and invaded plot^63^, as well as the alien phylogenetic relatedness to natives (ANMPD) for the invaded plots^64^. These phylogenetic matrices were calculated using the functions PD and MPD in the R package picante (1.8 version^65^).

To test whether the phenotypic resemblance of native species is affected by the presence of alien species, we quantified the phylogenetic signal in five phenotypic traits (height, biomass, leaf production, floral production, SLA) of the native species in each plot using Blomberg's K and pagel’s lambda for continuous traits^66^, using the Phylosignal function in the R package picante. For each plot, we used for each of the native species the trait value averaged across the measured individuals. Significance of the phylogenetic signal in each plot was estimated through 999 randomizations with the trait distribution randomly shuffled across phylogenetic tips.

To quantify the functional diversity in each plot, we used our five phenotypic traits to calculate multi-trait functional richness (FRic) and functional dispersion (FDis). FRic is the amount of functional space filled by the community, an analogue of trait range in a multidimensional space^61^. FRic is calculated as the pairwise functional dissimilarity across species, using the Euclidean distance in multi-trait space after standardizing each trait to a mean of zero and a standard deviation of one. FDis is the multidimensional trait space or mean distance of each species, weighted by its relative abundance, to the centroid of all species in a community^67,68^. FDis for each plot was calculated using function dbFD which is available in the FD package^67^ in R version 3.2.5 (R Development Core Team 2014).

In addition to the functional diversity of the native communities, we also quantified the alien functional relatedness (ANMFD) to native species as well as the native functional relatedness (NMFD), using the same set of traits as used for the calculation of FRic and FDis. ANMFD was calculated as the mean weighted (by abundance) pairwise Euclidian distance of each alien species to the native community, NMFD was calculated as the mean weighted pairwise Euclidian distance among natives within each pair plot^36,69^, using the ‘dist’ function in R (package ‘stats’ version 3.7.0).

### Comparing native diversity between invaded and non-invaded plots

To assess effects of the alien species on native plant diversity, we compared native species richness (SR), abundance, phylogenetic diversity indices (PD, NMPD), functional diversity indices (NMFD, FDis, FRic) of the invaded versus non-invaded plots using *t*-test for paired comparisons. To account for false discovery rates in the multiple comparisons, we applied Bonferroni correction for each test. We then used ANOVA (with Bonferroni correction) to test whether these native indices differed between invaded and non-invaded plots.

To visualize whether there was a difference in native species composition between invaded and non-invaded plots, we performed non-metric multidimensional scaling analysis (NMDS^70^) based on the Jaccard index of dissimilarity. To test whether the native species composition differed between the invaded and non-invaded plots, we used permutational analysis of variance (PERMANOVA, Anderson 2001). We performed these analyses using the *metaMDS*, and *adonis* functions of the Vegan package^71^.

To assess alien impact on species richness, abundance, FDis, FRic and PD of native communities, we calculated the changes in the different native diversity indices between the invaded and non-invaded plot of each pair using the following equation^72,73^ where *x* is diversity index of interest (richness, abundance, FDis, FRic, PD). So, for each index, we had 83 *Alien Impact*_*x*_ values, where positive values indicate an increase in the diversity index due to alien invasion, and negative *Alien Impact*_*x*_ values indicates a decline.

$$Alien\,Impact_{x} = \frac{{x_{{invaded\,communities}} ~ - ~x_{{non{\text{ - }}invaded\,communities}} ~}}{{x_{{invaded~\,communities}} ~ + ~x_{{non{\text{ - }}invaded\,communities}} }}$$

To test how *Alien Impact*_*x*_ depends on the resource availability and the similarity of the alien species to the native community, we constructed series of Generalized Additive Models (GAM). Here, *Alien Impact*_*x*_ for the different native diversity indices (SR, abundance, FDis, FRic, PD) were the response variables, and soil variables (nitrogen, organic carbon and moisture contents) and the dissimilarity between aliens and natives (ANMFD and ANMPD) were the explanatory variables. All models were compared using Akaike Information Criterion and Akaike weights to find an optimal model structure that best explains *Alien Impact*_*x*_^74^. We checked diagnostic plots (e.g. residuals versus fitted values and observed versus fitted values) to identify potential outliers, and whether the assumptions of homogeneity of variance and normality were not violated. In addition, we tested for normality of the residuals using the Shapiro–Wilk test^75^. To overcome the large spread of fitted values, phylogenetic measures were log transformed in order to improve the normality of the error distribution. All correlations between explanatory variables were < 0.6, indicating that multicollinearity should not be a problem. All analyses were done using R v.3.3.1 (RStudio Team 2018).

## Results

**Figure 1:**
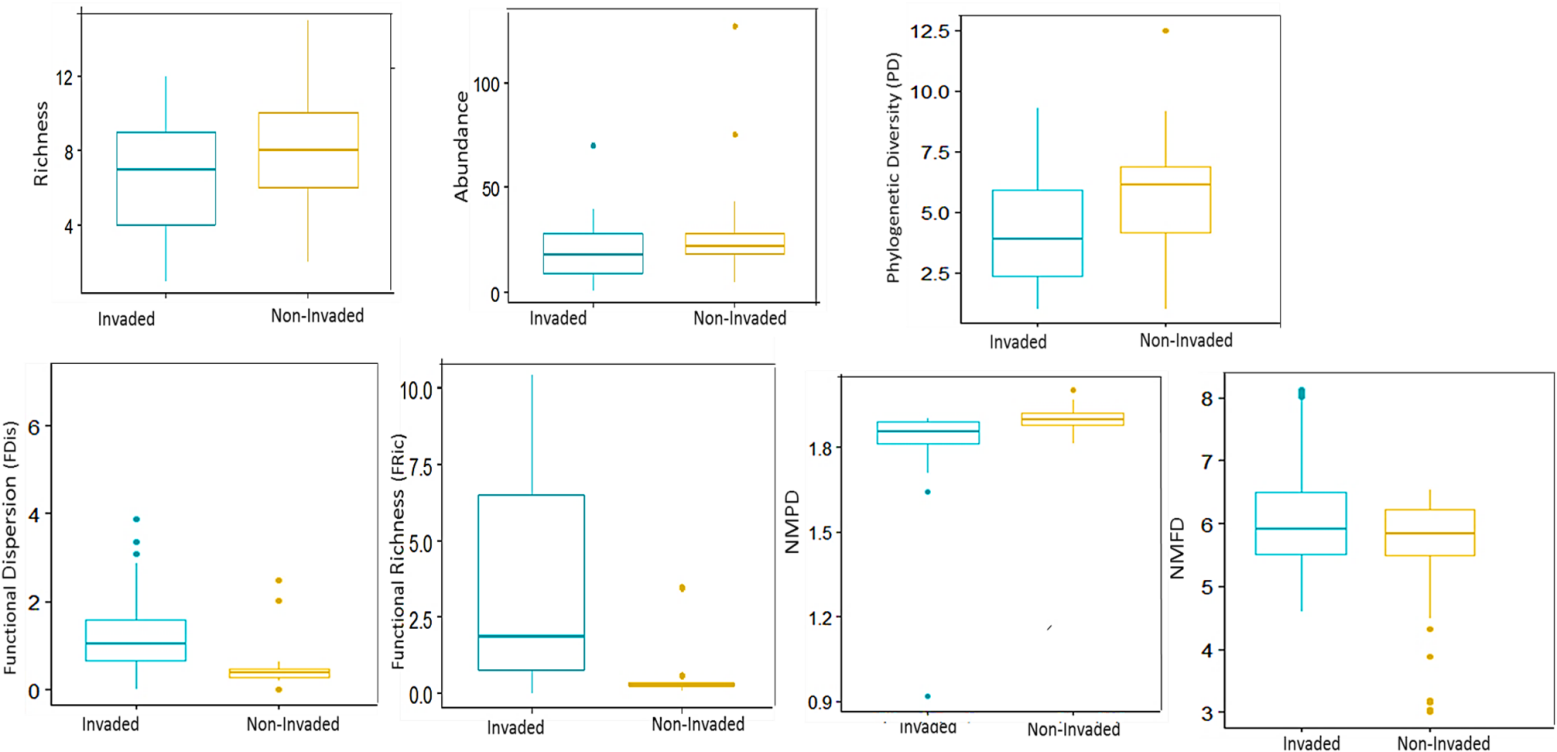
Mean differences in the native richness and abundance, phylogenetic Diversity (PD), Functional Dispersion (FDis), Functional richness (FRic), Native phylogenetic relatednes (NMPD), Native functional relatedness (NMFD) between invaded plots and non-invaded ones.

**Figure 2:**
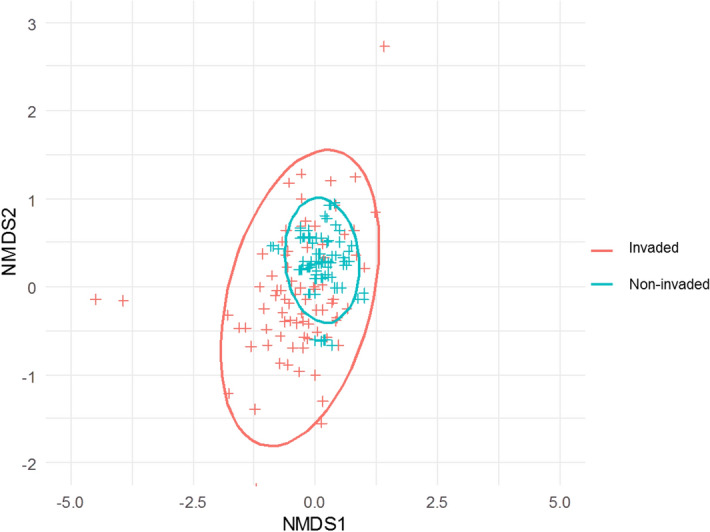
Non-metric multidimensional scaling analysis (NMDS) showing the differences in the native species composition between invaded plots and non-invaded ones (stress = 0.09 for the two axes). Red crosses represent invaded plots, blue crosses non-invaded ones. The colored ellipses are centered on the centroid of both groups and their size is proportional to the standard error of the coordinates of the corresponding points.

In total, 33 alien and 130 native plant species were recorded within the sampled plots, covering a gradient of soil resources. The two major findings are the following: first, comparisons between invaded and non-invaded plots revealed that native functional diversity indices (FDis, FRic) were significantly higher in invaded plots than non-invaded ones. Interestingly, we found an evidence that supports Darwin’s pre-adaptation hypothesis; native phylogenetic indices (PD, NMPD), richness and abundance were marginally lower in invaded plots compared to non-invaded ones (Table 1, Fig. 1). Additionally, there was a significant decline in the phylogenetic signals of native traits in invaded plots compared to non-invaded ones (Table 3). Second, aliens were both functionally and phylogenetically dissimilar to natives in richer environments; they had high positive impact on native richness, functional and phylogenetic diversity indices (SR, FD, FR, PD), indicating that the presence of aliens increased native diversity in such environments. By contrast, aliens were both functionally and phylogenetically similar to natives in limited resources environments; they had a significant negative impact on native diversity measures, indicating that presence of aliens was associated with negative effects on native diversity measures in such environments (Table 2, Fig. 2).

**Table 1:** Results of *t-test* for the differences in five native diversity metrics between non-invaded and invaded plots (mean and 95% confidence intervals, CI).

Response variables	Mean difference Z-Score	Lower CI	Upper CI	*p adj*
SR	1.497	0.892	2.101	0.6
Abundance	7.43	− 1.217	16.077	0.1
FDis	− 0.673	− 1.363	0.017	0.05
FRic	− 2.713	− 5.307	− 0.119	**0.03**
PD	1.557	0.266	2.848	**0.01**
NMPD	0.104	0.044	0.165	**0.001**
NMFD	− 183.986	− 267.629	− 100.344	**< 0.001**

SR is the number of native species within each plot, Abundance is the number of individuals, FDis is the functional dispersion, FRic is the functional richness, PD is the faith's phylogenetic diversity, NMPD, NMFD is the native phylogenetic and functional relatedness respectively. *p*-values were adjusted following Bonferroni approach, significant adjusted p-values are in boldface type.

**Table 2:** F-static, coefficients (Coef), and p-values predicted by GAM models that test the effects of soil resources (soil moisture—organic matter-nitrogen) represented by their PCA scores and the alien functional and phylogenetic relatedness to natives (ANMFD, ANMPD respectively) on alien impact (*AI*). Response variables are represented by *AI* on native abundance (NA), native species richness (SR), native functional diversity (FDis), Native Phylogenetic Diversity (PD) and Functional Richness (FRic); Note: *AI* was calculated as a ratio between the difference in the native diversity (between invaded plots and non-invaded plots), and the sum of native diversity of both plots type (see “Methods” section).

Explanatory variables	*AI* (NA)			*AI* (SR)			*AI* (FDis)			*AI* (PD)			*AI* (FRic)		
	Coef	F-static	*p* value	Coef	F-static	*p* value	Coef	F-static	*p* value	Coef	F-static	*p* value	Coef	F-static	*p* value
ANMFD	0.357	31.131	< 0.0001	0.361	7.65	< 0.001	0.631	57.577	< 0.0001	0.355	7.808	0.006	0.437	3.821	0.05
ANMPD	1.68	258.642	< 0.0001	1.547	14.491	< 0.001	2.52	35.649	< 0.0001	1.55	24.904	< 0.001	2.32	887.017	< 0.001
Soil Resources	0.232	23.083	0.006	0.237	9.295	< 0.001	0.437	3.754	0.001	0.238	24.214	< 0.001	0.261	0.007	0.93
Deviance explained (R^2^)		92.2%			86.4%			79.8%			82.6%			80.7%

### Relative differences in native diversity indices between invaded/non-invaded plots

In total, there were significant differences in native diversity between invaded and non-invaded plots, indicating significant effects of alien species on native diversity indices (Table 1, Fig. 1). Paired comparisons revealed that native richness and abundance were slightly lower (− 10.65% and − 9.83% respectively) in invaded plots compared to non-invaded plots (t-test, z = 1.496, *p* < 0.6) (Table 1). Native phylogenetic diversity and phylogenetic relatedness were marginally lower in invaded plots than in non-invaded ones (t-test, z = − 1.55, *p* < 0.05; t-test = − 1.78, *p* < 0.07) and declined by 9.7% and 10%, respectively, compared to non-invaded plots. However, native functional dispersion and functional richness were significantly higher in invaded plots compared to non-invaded ones (t- test, z = − 0.672, *p* < 0.01; z = − 2.713, *p* < 0.01 respectively) (Table 1, Fig. 1).

### Relative differences in native composition between invaded/non-invaded plots

The NMDS analysis and PERMANOVA test revealed statistically significant differences in native species composition between invaded and non-invaded plots. NMDS analysis (Fig. 2) showed that the non-invaded plots tended to have similar species composition and formed a subset of the invaded plots whose compositions were highly dispersed. PERMANOVA analyses confirmed this pattern by unravelling significant dispersion in the native species composition in invaded plots compared to non-invaded ones (R^2^ = 0.044, *F-value* = 5.18, *p* < 0.001, 999 permutations), which was influenced by the presence of alien species.

### Relative differences in the phylogenetic signal of traits of native species between invaded/non-invaded plots

There was a decline in the phylogenetic signal of measured native traits (height-biomass-leaf production-floral production-SLA) in invaded plots compared to non-invaded ones (Table 3). For shoot biomass, height and floral production of native species, the Blomberg’s K values declined significantly in invaded plots (K = 0.67, *p* < 0.01), but such decline was non-significant for leaf production and SLA. This indicates that the phylogenetic signal of native species traits in invaded plots was weaker than expected by a Brownian motion model for trait evolution. In parallel, Pagel’s lambda values of such native traits were significantly lower in invaded plots compared to non-invaded ones (Table 3).

**Table 3:** Differences in the phylogenetic signals of native traits between invaded plots and non-invaded plots. Blomberg’s K: 1 = Brownian motion (BM), 0 = random expectation; Pagel’s lambda: 1 = BM, 0 = random expectation; p-values: * < 0.05, ** < 0.01, ***0.001.

Trait	Pagel’s λ		Blomberg’s K	
	Invaded	Non-invaded	Invaded	Non-invaded
Height (cm)	4.26 × 10^–5^	1.22***	0.70	0.83*
Shoot Biomass (kg)	0.053	1.23***	0.64	0.78*
Leaf production	0.097	1.21**	0.71	0.76
Floral production	4.26 × 10^–5^	1.20***	0.56	0.77*
SLA (cm^2^ g^−1^)	0.14	0.12	0.66	0.67

### Magnitude of Alien Impact along soil resources gradients

**Figure 3:**
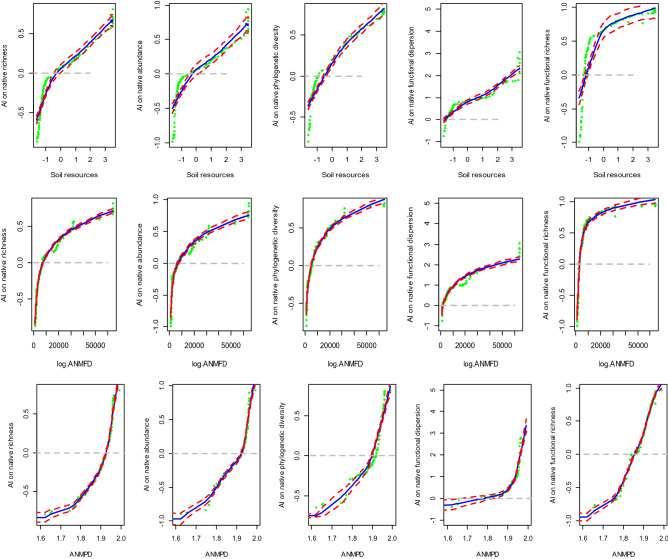
Alien impact (AI) on native richness, abundance, PD, FDis, FRic, in responding to soil resource availability (nitrogen, organic matter, soil moisture summarized in composite variable with a PCA) and the alien relatedness (functional and phylogenetic) to natives in SKP. Blue solid lines represent the average response expected by GAM model with 95% confidence intervals (red dashed lines). Green points represent the observed data points.

GAM models revealed that aliens tended to have significant positive impacts on native diversity indices in plots with high contents of soil organic carbon, nitrogen and moisture, but negative impacts in resource-limited plots (Table 2, Fig. 3). For native richness, abundance, functional dispersion, and native phylogenetic diversity, *Alien Impact*_*x*_ was positive in richer plots and negative in limited plots, with soil resources having highly significant effects (*F* = 23.08, *p* < 0.001; *F* = 9.30, *p* < 0.001; *F* = 3.75, *p* < 0.001; *F* = 24.21, *p* < 0.001, respectively). However, AI on native functional richness was non-significant and not affected by soil resources (*F* = 0.007, *p* < 0.93).

### Magnitude of Alien Impact along biotic relatedness

Alien species had significant positive impacts on native diversity measures, which increased with increasing functional and phylogenetic relatedness between aliens and natives (Table 2, Fig. 3). Aliens tended to have positive impacts on native diversity if they were functionally and phylogenetically dissimilar to natives; such impacts tended to be negative if aliens were more closely related to natives. For native richness and abundance, and native functional dispersion and richness, *Alien Impact*_*x*_ had positive effects with greater alien functional (ANMFD) and phylogenetic relatedness (ANMPD) to natives (*F* = 7.65, *p* < 0.0001; *F* = 31.13, *p* < 0.001;* F* = 57.577, *p* < 0.01; *F* = 35.649, *p* < 0.0001; *F* = 3.821, *p* < 0.001; *F* = 887.017, *p* < 0.001; *F* = 7.808, *p* < 0.001; F = 24.904, *p* < 0.001, respectively).

## Discussion

Documenting how alien species modify native community structure from a functional and phylogenetic point of view is a critical task to understanding the mechanisms driving alien impacts on native species assembly^6,7,29,76^. Interestingly, we found that invaded native communities are phylogenetically less, but functionally more diverse than non-invaded communities within our study area, indicating that the phylogenetic diversity of resident native communities can diverge from trait-based assembly processes. For example, in highly competitive environments, communities of phylogenetically under-dispersed species can be functionally over-dispersed to adapt to the intense competitive interactions^77^ among different functional species groups. Although, on average, the presence of alien plants affects the diversity components of invaded native communities, we highlight here that the magnitude of this impact strongly depends on resource availability and alien relatedness to natives.

First, our results support the hypothesis of niche-filling^26^, in which native species in invaded communities can be functionally more dissimilar to alien species and to each other than in non-invaded communities, which gives the possibility to alien species to contribute to the creation of gaps in the niche space by excluding functionally similar natives. The establishment of functionally-different native species is then possible, which would increase the functional dissimilarity of native communities^26,27,78^. By contrast, the presence of native species exploiting similar niches (i.e. intact native communities with filled niches) as the potential invader would confer high native biotic resistance due to the lack of an ‘empty niche’^79–81^, which would decrease the likelihood of establishment success even further. For example, this could happen when the spatial niche space is filled by productive native species that efficiently utilize local resources^82,83^ and occupy the same niche as the invader. However, this process at the functional level may not be mirrored by the phylogenetic diversity of native species, which in our case decreased in presence of aliens. Our results are consistent with the meta-analysis of Loiola et al., who proposed that aliens can fill empty gaps and occupy the existing functional niche space of the displaced species, rather than add novel evolutionary origins of resident native species^7,8,84^.

Ecological studies speculated that if the phylogenetic and functional components of invaded communities are concordant, the measured traits should exhibit a strong phylogenetic signal^85^. By contrast, our finding indicated an inconsistency between native PD and FD, which is supported by an observed decline in the phylogenetic signal of native species traits in the invaded communities compared to non-invaded ones. This inconsistency can thus be attributed to the fact that phylogenies are not completely capturing ecologically relevant traits. The weak phylogenetic signal for these traits could be due to their lack of phylogenetic conservatism or to convergent evolution in our study area^86^. Hence, although it is frequently assumed that the phylogenetic diversity is a surrogate for functional diversity in invasion studies^28,84,87^; but see, for example, Lososova et al., our results indicate that the phylogenetic structure can be complementary to, but not a substitute for, the functional trait structure^88^.

Indeed, the loss of native phylogenetic divergence in the invaded plots supports the prediction that competition can eliminate native species with conserved trait-based ecological strategies from phylogenetically-closely related taxa^31^. In other words, native species that have high phylogenetic convergence in their traits or similar trait values may decrease their potential advantage in competitive abilities^89^. For example, convergence in height may decrease the opportunity for individuals of taller species to outcompete those of smaller species in light‐limited environments^90^.

The inconsistency between phylogenetic under-dispersion and trait over-dispersion in invaded communities may also arise from the present-day strong negative interactions among phylogenetically related similar species (alien/native). This could lead to mutual exclusion of phylogenetically similar species and ultimately to divergence in trait space^77^. For example, highly competitive alien plants have a strong negative effect on native diversity, especially through increasing competition for resources such as light^91–95^. These interspecific interactions are particularly important for natives with more divergent traits, which are expected to be weakly conserved within phylogenetic lineages^77^.

We also observed a decline in native richness of invaded communities. A possible explanation of this change could be attributed to the covariation between environmental changes and presence of alien species^96–98^. For example, milder winters changed the environmental space of deciduous forests to conditions that are now more suitable for evergreen broad-leaved species^99^. Consequently, resident native species can become increasingly poorly adapted to the local environment, which will then provide opportunities for better-adapted aliens, ultimately declining the native richness through competitive exclusion.

Second, we found that aliens that were functionally and phylogenetically dissimilar to natives in resources rich environments^100^ are likely to expand the functional and phylogenetic space of native community (see Refs.^29,101,102^. This expansion may be attributed to the differences in the competitive abilities of both species groups^31,34,103^. For example, aliens with superior competitive ability tend to eliminate resident natives that have similar trait values as well as similar resources preferences. Thereby, if both species groups compete for different resources, and aliens outcompete resident natives, this should promote only dissimilar natives to co-exist with aliens^18,104^.

By contrast, we found that aliens functionally and phylogenetically similar to natives in resource-limited environments^100^ are expected to occupy a portion of the native functional space, leading to a reduction in native functional diversity. Previous studies (e.g. Refs.^105–107^) have suggested that both species groups should be more similar under stressful conditions^108,109^. This similarity is due to the strong effect of the environmental filtering that select species sharing analogous responses to the limiting environmental resources, a pattern that is consistent with the pre-adaptation hypothesis (PAH)^12,17^ even though these classic theories do not include facilitation as a potential mechanism. Eventually, alien species are presumed to occupy a portion of the functional space originally occupied by native species, leading to an increased functional similarity between both species groups^24,25^ and a reduction in native richness and subsequently in their functional and phylogenetic space.

## Conclusion

The presented paired-design empirical study highlighted that alien plants pose significant impacts at the native community structure^1^, and that such impacts are significantly linked to soil resources availability and to alien relatedness with resident natives. Significantly, we found evidence for the role of environmental filtering (pre-adaptation hypothesis) that regulate alien impacts on resident natives in harsh environments, while niche gap filling (Darwin’s Naturalization Hypothesis DNH) was the main mechanism determining such impacts in rich-resources environments. Our quantitative approach to value alien impacts could be further developed as the basis for underscoring alien species and recipient ecosystems for risk assessment of invasions^110^. We hope this contribution helps to invigorate this area of research by highlighting the association between invasion impacts and biotic and abiotic gradients at several levels of ecological complexity. As such, future studies should conduct similar ecological surveys in different ecosystems at fine and coarse scales to test the generality of our findings. For example, arid disturbed-invaded ecosystems generally undergo rapid human-driven disturbances that can strongly change the species composition similarity^111^, and thereby affect significantly the relatedness between alien and native groups. Further, It would be interesting to test the roles of different nutrient‐niches and biotic relatedness between aliens and natives on the magnitude of alien impacts, which might be an effective strategy for a global risk assessment of plant invasions.

## Supplementary Information

Supplementary Information.
