# Supplementary material for: Contrasting alien effects on native diversity along biotic and abiotic gradients in an arid protected area
Source: Sci Rep. 2021 Jun 30;11:13557. doi: 10.1038/s41598-021-92763-2 (PMC8245551; doi:10.1038/s41598-021-92763-2)
Supplement: Supplementary file 1 — Supplementary Information. [file 41598_2021_92763_MOESM1_ESM.docx]

**Contrasting alien effects on native diversity along biotic and abiotic gradients in an arid protected area**

**Reham F. El-Barougy^1,2*^, Ibrahim A. El gamal^3^, Abdel-Hamid A. Khedr^1^,** **Louis-Félix Bersier^2^**

*^1^ Department of Botany and Microbiology, Faculty of Science, Damietta University, Egypt*

*^2^Department of Biology, Ecology and Evolution , University of Fribourg, Avenue de l'Europe 20, 1700 Fribourg, Switzerland*

*^3^Nature Conservation Sector, Egyptian Environmental Affairs Agency, Cairo 11728, Egypt*

* Corresponding author: [reham.elbarougy@unifr.ch](mailto:reham.elbarougy@unifr.ch)

[Reham_fekry2012@du.edu.eg](mailto:Reham_fekry2012@du.edu.eg)

**Supplementary Material& Methods**

**Study area characteristics**

The study area was conducted in Saint Katherine Protectorate (SKP), South Sinai, Egypt (Fig. 1A), from March to July 2018 at the peak of the flowering season (Danin 2006). Saint Katherine Protectorate (SKP) was declared in 1996 as full protected-area status was given to approximately 4,350km² of largely mountainous terrain in South Sinai, but the studied area was given to approximately **100 – 150 km2**. The area includes the highest peaks in Egypt and contains a unique assemblage of natural resources, notably high-altitude ecosystems with surprisingly diverse fauna and flora and with a significant proportion of endemic species. The high mountains (1600–2460m asl) surrounding the town of St Katherine receive higher levels of precipitation, of up to 100mm per year (Ayyad et al. 2000).


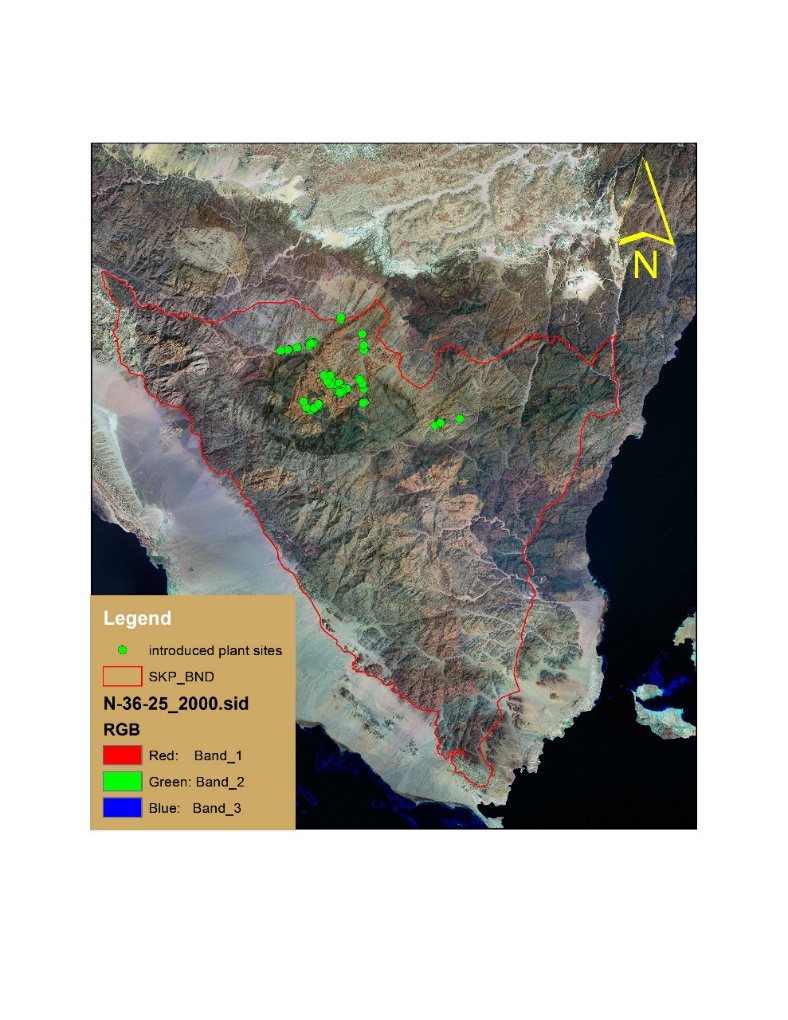
**Climate**

The Saint Katherine Protectorate lies in the arid North African belt and is characterized by a Saharan-Mediterranean climate, experiencing extremely dry, hot summers and cold winters. Average rainfall is 57mm a year, with maximum temperatures of 35 ◦C and lows of 5 ◦C. The high mountains (1600–2460m asl) surrounding the town of St Katherine receive higher levels of precipitation, of up to 100mm per year^1^. SKP encompasses approximately 4,350 km² of largely mountainous terrain in the South Sinai Governorate, but the study area was approximately 100–150 km^2^ in size.

**Native destructive trait data**

**Fig.1A**. Showed the study sites in SKP, drawn by I.A. Elgamal using ArcGIS software, version 10.4.1.ESRI (2011) ArcGIS Desktop: Release 10. Environmental Systems Research Institute, Redlands.

As destruction of native biodiversity is strictly forbidden within the SKP, we could not harvest the leaves or any other part of the native species. Therefore, we measured the total leaf area of the plants, by first drawing the outlines of their leaves on paper, and then measuring the areas of these leaf copies. We then measured the same traits, as well as SLA and aboveground biomass, on individuals of the native species that grew outside the protected areas. Then for each native species, we used multiple regression models to determine allometric equations ^2^ for SLA and aboveground biomass as functions of the non-destructive measurements. We then used these allometric equations, and the non-destructive measurements that we took in the SKP plots to estimate the SLA and aboveground biomass of the native plants there.

**Fig.A** Rooted ultrametric phylogenetic tree including the pool of 130 native species and 33 alien species collected from 83 pairs of invaded and non-invaded plots in Saint Katherine Protectorate.

**Table A** List of the 130 native and 33 alien plants included in the ultra metric tree with mean trait values and SD per species that were recorded in 83 pairs of plots were randomly placed throughout the study area in SKP

| Species Status | Family | Scientific name | Number of individuals | Height(cm) | | Biomass(kg) | | SLA(cm2/g) | | Floral Production | | Leaf Production | |
| --- | --- | --- | --- | --- | --- | --- | --- | --- | --- | --- | --- | --- | --- |
|  |  |  |  | Mean | SD | Mean | SD | Mean | SD | Mean | SD | Mean | SD |
| Alien | Fabaceae | *Acacia_saligna* | 34 | 463.88 | 268.28 | 75.55 | 64.41 | 42.425 | 12.127 | 309.38 | 509.23 | 8694.4 | 9619.3 |
| Alien | Agavaceae | *Agave_americana* | 7 | 131.66 | 51.071 | 2.66 | 0.57 | 3.672 | 0.0829 | 8.5 | 2.5 | 80 | 36.05 |
| Alien | Liliaceae | *Aloe_vera* | 16 | 87 | 78.693 | 9 | 7.118 | 4.1945 | 0.2442 | 9.25 | 18.5 | 77.5 | 50.58 |
| Alien | Amaranthaceae | *Amaranthus_caudatus* | 45 | 38.333 | 14.841 | 0.333 | 0.337 | 48.498 | 10.889 | 113.16 | 169.09 | 28.333 | 11.690 |
| Alien | Meliaceae | *Azadirachta_indica* | 9 | 60 | 0 | 5 | 0 | 238.88 | 0 | 5 | 0.5 | 20 | 3 |
| Alien | Chenopodiaceae | *Beta_vulgaris* | 256 | 42.5 | 31.819 | 35 | 35.35 | 136.35 | 3.9614 | 5.5 | 9.4604 | 11.167 | 2.0412 |
| Alien | Theaceae | *Camellia_sinensis* | 85 | 38.2 | 34.2593 | 1.48 | 1.0257 | 102.223 | 19.2305 | 20.2 | 27.225 | 48 | 32.9014 |
| Alien | Casuarinaceae | *Casuarina_cunninghamiana* | 3 | 700 | 424.264 | 80 | 56.569 | 52.9211 | 4.11685 | 500 | 707.107 | 1500 | 707.107 |
| Alien | Cactaceae | *Cereus_triangularis* | 5 | 101 | 140.007 | 27 | 4.2426 |  |  | 6 | 8.48528 | 95 | 7.07107 |
| Alien | Iridaceae | *Crocus_sativus* | 46 | 10 | 5 | 0.0533 | 0.0451 | 133.827 | 172.797 | 4.33333 | 5.1316 | 22.667 | 23.6714 |
| Alien | Asteraceae | *Cynara_cardunculus* | 1 | 60 | 0 | 4 | 0 | 32.6667 | 0 | 0 | NA | 15 | NA |
| Alien | Myrtaceae | *Eucalyptus_globulus* | 55 | 1889.23 | 2864.22 | 144.69 | 147.84 | 39.7354 | 12.7323 | 308.923 | 577.662 | 9076.9 | 8969.03 |
| Alien | Verbenaceae | *Lantana_camara* | 3 | 123 | 0 | 5 | 0 | 67.5 | 0 | 690 | NA | 780 | NA |
| Alien | Lamiaceae | *Mentha_longifolia* | 420 | 71.7 | 35.2611 | 4.25 | 3.7361 | 231.165 | 66.1297 | 206.778 | 421.227 | 97.222 | 78.7457 |
| Alien | Lamiaceae | *Mentha_spicata* | 128 | 15.1429 | 5.72796 | 0.4643 | 0.3326 | 132.482 | 106.697 | 27.5714 | 33.585 | 36.286 | 12.5129 |
| Alien | Aizoaceae | *Mesembryanthemum_acinaciforme* | 10 | 10 | 0 | 25 | 0 | 297.423 | 0 | 0 | NA | 1200 | NA |
| Alien | Moringaceae | *Moringa_oleifera* | 1 | 90 | 0 | 0.5 | 0 | 147.632 | 0 | 0 | NA | 17 | NA |
| Alien | Scrophulariaceae | *Myoporum_laetum* | 3 | 450 | 0 | 45 | 0 |  |  | 0 | NA | 1800 | NA |
| Alien | Apocynaceae | *Nerium_oleander* | 39 | 120.313 | 53.1184 | 8.75 | 9.6575 | 30.3692 | 3.03938 | 54.875 | 123.092 | 541.88 | 349.937 |
| Alien | Solanaceae | *Nicotiana_rustica* | 596 | 60 | 22.7303 | 36.75 | 41.724 | 114.087 | 49.3916 | 14.5 | 14.1774 | 61.25 | 27.1953 |
| Alien | Cactaceae | *Opuntia_ficus.indica* | 2 | 67.5 | 3.53553 | 12 | 0 | 2.9372 | 0.13449 | 16.5 | 12.0208 | 400 | 70.7107 |
| Alien | Lamiaceae | *Origanum_majorana* | 31 | 27 | 13.9403 | 0.9786 | 0.7852 | 126.341 | 28.5136 | 292.857 | 753.01 | 831.29 | 591.609 |
| Alien | Papaveraceae | *Papaver_somniferum* | 12 | 61.1667 | 25.6548 | 0.2477 | 0.2123 | 73.4351 | 3.10039 | 2.33333 | 1.50555 | 21.5 | 14.2092 |
| Alien | Geraniaceae | *Pelargonium_peltatum* | 4 | 67.5 | 74.2462 | 1.5 | 0.7071 | 40.2441 | 4.77647 | 30 | 28.2843 | 55 | 7.07107 |
| Alien | Anacardiaceae | *Pistacia_lentiscus* | 9 | 301.667 | 207.019 | 33.333 | 28.925 | 84.1039 | 56.5562 | 258.333 | 412.816 | 7750 | 8489.7 |
| Alien | Lamiaceae | *Plectranthus_hadiensis* | 200 | 60 | 0 | 80 | 0 | 140.888 | 0 | 160 | NA | 900 | NA |
| Alien | Anacardiaceae | *Rhus_coriaria* | 6 | 187.5 | 53.033 | 17.5 | 10.607 | 22.1266 | 6.45183 | 172.5 | 180.312 | 2500 | 707.107 |
| Alien | Rosaceae | *Rosa_gallica* | 15 | 93.6 | 32.7624 | 1.6 | 1.022 | 79.9454 | 38.2805 | 45.8 | 91.254 | 123.5 | 136.057 |
| Alien | Lamiaceae | *Rosmarinus_officinalis* | 271 | 83.4444 | 56.9813 | 23.154 | 33.798 | 39.6238 | 21.5371 | 163.111 | 306.811 | 13930 | 16706.5 |
| Alien | Lamiaceae | *Salvia_fruticosa* | 93 | 64.3571 | 31.5707 | 3.9929 | 4.6076 | 125.397 | 128.638 | 21.5 | 30.8788 | 1311.9 | 1667.04 |
| Alien | Simmondsiaceae | *Simmondsia_chinensis* | 1 | 190 | 0 | 50 | 0 | 29 | 0 | 80 | NA | 20000 | NA |
| Alien | Cupressaceae | *Thuja_orientalis* | 1 | 500 | 0 | 30 | 0 | 408.333 | 0 | 0 | NA | 900 | NA |
| Alien | Agavaceae | *Yucca_gloriosa* | 9 | 172.5 | 48.5541 | 9.6667 | 10.671 | 145.295 | 20.4763 | 90 | 201.494 | 156.5 | 115.523 |
| Native | Asteraceae | *Achillea_fragrantissima* | 91 | 51.0192 | 29.0628 | 1.6 | 0.4449 | 1.59998 | 0.44487 | 84.75 | 136.268 | 411.44 | 231.21 |
| Native | Fabaceae | *Alhagi_graecorum* | 3 | 40 | 0 | 0.7157 | 0 | 0.71572 | 0 | 6 | NA | 200 | NA |
| Native | Boraginaceae | *Alkanna_orientalis* | 30 | 18 | 17.9782 | 1.7223 | 1.2954 | 1.50356 | 0.47021 | 66.5882 | 112.851 | 352.53 | 252.031 |
| Native | Chenopodiaceae | *Anabasis_articulata* | 15 | 31 | 9.66954 | 1.59 | 0.2077 | 1.58998 | 0.20774 | 20.6667 | 9.99333 | 140 | 32.8634 |
| Native | Primulaceae | *Anagallis_arvensis* | 10 | 10 | 0 | 1.2918 | 0 | 1.29184 | 0 | 32 | NA | 50 | NA |
| Native | Scrophulariaceae | *Anarrhinum_pubescens* | 10 | 21 | 21.9393 | 1.3259 | 0.1453 | 1.32591 | 0.1453 | 59.5 | 41.7193 | 60 | 70.7107 |
| Native | Boraginaceae | *Anchusa_milleri* | 20 | 7.25 | 2.05287 | 1.3506 | 0.0986 | 1.35057 | 0.09861 | 6 | 4.54606 | 13 | 2.44949 |
| Native | Asteraceae | *Artemisia_judaica* | 93 | 75.5161 | 31.9049 | 1.9003 | 0.4204 | 1.90029 | 0.42043 | 125.182 | 253.466 | 1752.4 | 1101.69 |
| Native | Poaceae | *Arundo_donax* | 40 | 450 | 57.735 | 1.5266 | 0.4171 | 1.5266 | 0.41705 | 21.5 | 2.12132 | 46.5 | 4.94975 |
| Native | Fabaceae | *Astragalus_spinosus* | 2 | 188.667 | 269.623 | 2.2192 | 0.002 | 2.2192 | 0.002 | 12.5 | 10.6066 | 585 | 756.604 |
| Native | Fabaceae | *Astragalus_tribuloides* | 16 | 2.66667 | 0.7785 | 1.1989 | 0.4663 | 1.19886 | 0.46629 | 6.66667 | 4.84424 | 34.667 | 10.9484 |
| Native | Asteraceae | *Atractylis_carduus* | 1 | 4 | 0 | 1.4417 | 0 | 1.44167 | 0 | 1 | NA | 23 | NA |
| Native | Poaceae | *Avena_barbata* | 17 | 76.5 | 7.50555 | 1.2978 | 0.1144 | 1.29784 | 0.1144 | 31 | 41.0122 | 23 | 4.24264 |
| Native | Poaceae | *Avena_fatua* | 10 | 130 | 0 | 1.6201 | 0 | 1.6201 | 0 | 33 | NA | 30 | NA |
| Native | Lamiaceae | *Ballota_undulata* | 7 | 36.5 | 14.4815 | 1.6498 | 0.213 | 1.64981 | 0.21295 | 15.8 | 8.31865 | 345.8 | 73.5405 |
| Native | Acanthaceae | *Blepharis_attenuata* | 1 | 7 | 0 | 1.5921 | 0 | 1.59208 | 0 | 13 | NA | 120 | NA |
| Native | Poaceae | *Brachypodium_distachyon* | 23 | 16.6667 | 5.16398 | 1.6475 | 0.4196 | 1.64746 | 0.41958 | 12.6667 | 13.0128 | 27.667 | 6.80686 |
| Native | Poaceae | *Bromus_pectinatus* | 24 | 21.6 | 10.4785 | 1.9002 | 0.1448 | 1.90025 | 0.14481 | 21.75 | 24.3088 | 9.5 | 1 |
| Native | Resedaceae | *Caylusea_hexagyna* | 15 | 106.1 | 314.086 | 1.779 | 0.4683 | 1.77905 | 0.46828 | 11.1429 | 11.0518 | 172.57 | 365.002 |
| Native | Asteraceae | *Centaurea_scoparia* | 10 | 36.6 | 17.2569 | 2.1118 | 0.3701 | 2.11178 | 0.37013 | 175.75 | 303.213 | 43.5 | 24.8395 |
| Native | Asteraceae | *Centaurea_sinaica* | 3 | 15 | 0 | 1.8967 | 0 | 1.89669 | 0 | 3 | NA | 16 | NA |
| Native | Chenopodiaceae | *Chenopodium_album* | 45 | 16.5652 | 6.83458 | 1.2114 | 0.6023 | 1.21142 | 0.6023 | 148.667 | 333.629 | 22 | 14.1164 |
| Native | Chenopodiaceae | *Chenopodium_murale* | 8 | 15.5 | 0.57735 | 0.5731 | 0.3234 | 0.57306 | 0.32335 | 49 | 1.41421 | 13 | 1.41421 |
| Native | Asteraceae | *Chiliadenus_montanus* | 13 | 22.1429 | 2.67261 | 1.5301 | 0.7164 | 1.53011 | 0.71636 | 39.5 | 55.9434 | 955 | 981.648 |
| Native | Cleomiaceae | *Cleome_arabica* | 30 | 71.1818 | 23.0687 | 1.8066 | 0.3991 | 1.80659 | 0.39911 | 20.1111 | 20.8713 | 209.78 | 73.7746 |
| Native | Cleomiaceae | *Cleome_droserifolia* | 3 | 14 | 0 | 1.6495 | 0 | 1.64953 | 0 | 49 | NA | 345 | NA |
| Native | Lamiaceae | *Clinopodium_serpyllifolium* | 1 | 80 | 0 | 1.9626 | 0 | 1.96264 | 0 | 19 | NA | 123 | NA |
| Native | Fabaceae | *Colutea_istria* | 5 | 214 | 8.94427 | 1.4851 | 0.2913 | 1.4851 | 0.29134 | 96 | 125.491 | 1005.3 | 406.356 |
| Native | Asteraceae | *Conyza_aegyptiaca* | 22 | 31 | 13.5056 | 1.504 | 0.2777 | 1.50397 | 0.2777 | 2 | 3.4641 | 59.333 | 23.029 |
| Native | Asteraceae | *Conyza_bonariensis* | 4 | 37 | 3.4641 | 1.0724 | 0.2539 | 1.07243 | 0.25394 | 28 | 16.9706 | 36 | 8.48528 |
| Native | Cucurbitaceae | *Cucumis_prophetarum* | 2 | 3 | 0 | 1.8916 | 0 | 1.89162 | 0 | 7 | NA | 110 | NA |
| Native | Solanaceae | *Datura_ferox* | 1 | 20 | 0 | 0.6555 | 0 | 0.65553 | 0 | 2 | NA | 10 | NA |
| Native | Solanaceae | *Datura_stramonium* | 1 | 20 | 0 | 0.8338 | 0 | 0.83376 | 0 | 1 | NA | 16 | NA |
| Native | Poaceae | *Digitaria_sanguinalis* | 10 | 58.6667 | 6.35085 | 1.3078 | 0.711 | 1.777 | 0.71104 | 36.5 | 26.163 | 48 | 2.82843 |
| Native | Brassicaceae | *Diplotaxis_acris* | 39 | 36.208 | 20.792 | 1.484 | 0.303 | 1.4841 | 0.3034 | 80.071 | 264.84 | 45.071 | 41.332 |
| Native | Brassicaceae | *Diplotaxis_harra* | 10 | 17.5 | 8.6602 | 1.158 | 0.049 | 1.1581 | 0.0492 | 9 | 4.2426 | 12.5 | 6.3639 |
| Native | Asteraceae | *Echinops_glaberrimus* | 2 | 150 | 0 | 1.542 | 0 | 1.5428 | 0 | 30 | NA | 87 | NA |
| Native | Asteraceae | *Echinops_spinosus* | 5 | 45.571 | 44.664 | 2.1888 | 0.473 | 2.1887 | 0.47382 | 526.75 | 683.176 | 28.5 | 14.5258 |
| Native | Geraniaceae | *Erodium_laciniatum* | 4 | 3.175 | 0.2362 | 1.3631 | 0.301 | 1.3630 | 0.3011 | 6.6666 | 4.7258 | 19.667 | 4.1633 |
| Native | Brassicaceae | *Eruca_sativa* | 4 | 25 | 0 | 1.231 | 0 | 1.2309 | 0 | 10 | NA | 23 | NA |
| Native | Euphorbaiceae | *Euphorbia_hirta* | 5 | 4 | 0 | 1.070 | 0 | 1.0708 | 0 | 10 | NA | 64 | NA |
| Native | Euphorbaiceae | *Euphorbia_obovata* | 3 | 10 | 0 | 1.509 | 0 | 1.5992 | 0 | 3 | NA | 120 | NA |
| Native | Euphorbaiceae | *Euphorbia_peplus* | 10 | 10 | 0 | 2.048 | 0 | 2.0483 | 0 | 4 | NA | 25 | NA |
| Native | Euphorbaiceae | *Euphorbia_retusa* | 7 | 26.5 | 4.7258 | 1.820 | 0.064 | 1.8203 | 0.0643 | 14 | 12.288 | 316.67 | 106.9 |
| Native | Zygophyllaceae | *Fagonia_arabica* | 34 | 14.84 | 9.7625 | 1.872 | 1.293 | 1.6180 | 0.2062 | 14.882 | 7.9990 | 70.294 | 32.215 |
| Native | Zygophyllaceae | *Fagonia_bovie* | 3 | 30 | 0 | 1.558 | 0.067 | 1.5586 | 0.0767 | 20.5 | 19.091 | 117 | 8.4852 |
| Native | Zygophyllaceae | *Fagonia_bruguieri* | 1 | 10 | 0 | 2.952 | 0 | 2.9527 | 0 | 50 | NA | 67 | NA |
| Native | Zygophyllaceae | *Fagonia_mollis* | 95 | 15.018 | 6.2494 | 1.8355 | 0.905 | 1.7202 | 0.3613 | 57.368 | 157.24 | 146.68 | 77.921 |
| Native | Brassicaceae | *Farsetia_aegyptia* | 2 | 34.666 | 2.8867 | 2.359 | 0.630 | 2.3597 | 0.6304 | 31.5 | 2.1213 | 167 | 15.556 |
| Native | Moraceae | *Ficus_carica* | 1 | 100 | 0 | 1.738 | 0 | 1.7380 | 0 | 100 | NA | 560 | NA |
| Native | Moraceae | *Ficus_palmata* | 6 | 135 | 65.366 | 1.744 | 0.35 | 1.7440 | 0.3583 | 98 | 127.7 | 842.17 | 273.98 |
| Native | Apiaceae | *Foeniculum_vulgare* | 20 | 62.9 | 58.465 | 1.467 | 0.260 | 1.4675 | 0.2606 | 111.5 | 281.68 | 81.8 | 58.913 |
| Native | Urticaceae | *Forsskaolea_tenacissima* | 3 | 100.66 | 94.685 | 1.562 | 0.134 | 1.5622 | 0.1343 | 39.5 | 7.7781 | 77.5 | 45.961 |
| Native | Asteraceae | *Glebionis_coronaria* | 2 | 17 | 0 | 1.052 | 0 | 1.0526 | 0 | 80 | NA | 46 | NA |
| Native | Chenopodiaceae | *Haloxylon_salicornicum* | 5 | 50 | 0 | 2.004 | 0 | 2.004 | 0 | 0 | NA | 346 | NA |
| Native | Boraginaceae | *Heliotropium_arbainense* | 10 | 12 | 2.4899 | 1.327 | 0.286 | 1.3274 | 0.2868 | 25.5 | 30.051 | 131.67 | 39.256 |
| Native | Poaceae | *Hordeum_marinum* | 107 | 10.437 | 3.4635 | 1.412 | 0.629 | 1.4124 | 0.6296 | 24 | 15.362 | 27.818 | 50.528 |
| Native | Solanaceae | *Hyoscyamus_boveanus* | 2 | 24.5 | 1.732 | 1.536 | 0.416 | 1.5368 | 0.4169 | 20 | 14.142 | 306 | 114.55 |
| Native | Solanaceae | *Hyoscyamus_pusillus* | 1 | 7 | 0 | 1.712 | 0 | 1.7011 | 0 | 400 | NA | 30 | NA |
| Native | Asteraceae | *Iphiona_mucronata* | 4 | 14.666 | 2.8867 | 1.861 | 0.227 | 1.8616 | 0.2269 | 11.5 | 16.263 | 380.5 | 44.547 |
| Native | Asteraceae | *Iphiona_scabra* | 5 | 15 | 0 | 1.965 | 0 | 1.9650 | 0 | 12 | NA | 120 | NA |
| Native | Juncaceae | *Juncus_bufonius* | 5 | 63 | 0 | 1.975 | 0 | 1.9750 | 0 | 116 | NA | 25 | NA |
| Native | Asteraceae | *Lactuca_serriola* | 3 | 46 | 0 | 1.5278 | 0 | 1.52782 | 0 | 0 | NA | 23 | NA |
| Native | Lamiaceae | *Lamium_amplexicaule* | 7 | 14 | 0 | 0.312 | 0 | 0.31199 | 0 | 3 | NA | 56 | NA |
| Native | Asteraceae | *Launaea_nudicaulis* | 2 | 8.66667 | 1.1547 | 1.7777 | 0.0296 | 1.77775 | 0.02957 | 48.5 | 30.4056 | 17.5 | 3.53553 |
| Native | Asteraceae | *Launaea_spinosa* | 5 | 21.25 | 8.09835 | 1.5337 | 0.086 | 1.53368 | 0.08601 | 47.3333 | 31.0054 | 93.333 | 11.7189 |
| Native | Lamiaceae | *Lavandula_coronopifolia* | 1 | 90 | 0 | 1.3778 | 0 | 1.37784 | 0 | 0 | NA | 458 | NA |
| Native | Asteraceae | *Leysera_leyseroides* | 4 | 14 | 0 | 1.8091 | 0.0664 | 1.80905 | 0.06643 | 19 | 1.41421 | 140 | 0 |
| Native | Plumbaginaceae | *Limonium_lobatum* | 2 | 10 | 0 | 1.5322 | 0 | 1.53219 | 0 | 30 | NA | 27 | NA |
| Native | Plumbaginaceae | *Limonium_pruinosum* | 7 | 21.6 | 3.20936 | 1.3975 | 0.2753 | 1.39752 | 0.27527 | 29.6667 | 2.88675 | 32.667 | 1.1547 |
| Native | Plumbaginaceae | *Limonium_sinuatum* | 2 | 35 | 0 | 1.2017 | 0 | 1.20168 | 0 | 5 | NA | 40 | NA |
| Native | Fabaceae | *Lotononis_platycarpa* | 1 | 2 | 0 | 1.1584 | 0 | 1.1584 | 0 | 10 | NA | 40 | NA |
| Native | Solanaceae | *Lycium_shawii* | 1 | 110 | 0 | 1.6787 | 0 | 1.67868 | 0 | 24 | NA | 560 | NA |
| Native | Malvaceae | *Malva_neglecta* | 39 | 6.30769 | 2.2763 | 1.2083 | 0.5665 | 1.20829 | 0.56653 | 135.923 | 428.503 | 10.846 | 3.78255 |
| Native | Malvaceae | *Malva_parviflora* | 3 | 12 | 0 | 1.3242 | 0 | 1.32423 | 0 | 1 | NA | 12 | NA |
| Native | Brassicaceae | *Matthiola_arabica* | 17 | 28.0909 | 12.6527 | 2.3438 | 1.8892 | 1.77138 | 0.2245 | 17.5556 | 11.7804 | 256.44 | 160.604 |
| Native | Brassicaceae | *Matthiola_longipetala* | 37 | 42.5294 | 13.7027 | 1.6439 | 0.668 | 1.64392 | 0.66797 | 47.2308 | 32.1511 | 370.69 | 138.207 |
| Native | Brassicaceae | *Morettia_canescens* | 3 | 3.16667 | 0.28868 | 1.8912 | 0.2236 | 1.89119 | 0.22356 | 11.5 | 16.2635 | 127 | 9.89949 |
| Native | Solanaceae | *Nicotiana_glauca* | 1 | 160 | 0 | 1.2486 | 0 | 1.2486 | 0 | 1450 | NA | 223 | NA |
| Native | Resedaceae | *Ochradenus_baccatus* | 20 | 85 | 27.356 | 1.6639 | 0.1834 | 1.66387 | 0.18342 | 176.818 | 389.435 | 472.27 | 126.292 |
| Native | Oleaceae | *Olea_europaea* | 20 | 137.5 | 58.6405 | 1.6698 | 0.2523 | 1.66978 | 0.25234 | 95.5 | 306.366 | 1782 | 916.358 |
| Native | Resedaceae | *Oligomeris_linifolia* | 3 | 53.3333 | 40.4145 | 2.2505 | 0.1337 | 2.25053 | 0.13365 | 17.5 | 24.7487 | 1005 | 1251.58 |
| Native | Asteraceae | *Onopordum_ambiguum* | 6 | 76 | 42.7785 | 1.6707 | 0.1377 | 1.67069 | 0.13771 | 6.66667 | 5.7735 | 12.333 | 3.78594 |
| Native | Lamiaceae | *Origanum_syriacum* | 214 | 21 | 16.3818 | 1.8604 | 0.7867 | 1.86035 | 0.78669 | 5.66667 | 7.9666 | 206 | 119.558 |
| Native | Orobanchaceae | *Orobanche_palaestina* | 3 | 30 | 0 | 0.2816 | 0 | 0.28164 | 0 | 0 | NA | 0 | NA |
| Native | Oxalidaceae | *Oxalis_corniculata* | 10 | 23 | 0 | 2.1024 | 0.4425 | 2.1024 | 0.4425 | 19.5 | 14.8492 | 31.5 | 4.94975 |
| Native | Papaveraceae | *Papaver_decaisnei* | 3 | 30 | 0 | 1.517 | 0 | 1.51705 | 0 | 4 | NA | 30 | NA |
| Native | Zygophyllaceae | *Peganum_harmala* | 21 | 29.9048 | 18.1959 | 1.751 | 0.3827 | 1.75095 | 0.38267 | 41.1538 | 30.8217 | 409.69 | 204.104 |
| Native | Lamiaceae | *Phlomis_aurea* | 3 | 128.333 | 14.4338 | 1.7911 | 0.0103 | 1.79111 | 0.01034 | 95.5 | 40.3051 | 585 | 120.208 |
| Native | Poaceae | *Phragmites_australis* | 10 | 134 | 0 | 1.6631 | 0 | 1.66311 | 0 | 500 | NA | 34 | NA |
| Native | Plantagonaceae | *Plantago_sinaica* | 9 | 14.2 | 3.83406 | 1.613 | 0.0855 | 1.61296 | 0.08554 | 129 | 62.3538 | 330 | 95.3939 |
| Native | Portulacaceae | *Portulaca_oleracea* | 13 | 20 | 4.47214 | 1.2302 | 0.1448 | 1.2302 | 0.14476 | 17.6667 | 24.0069 | 39.333 | 6.02771 |
| Native | Asteraceae | *Pulicaria_incisa* | 7 | 21 | 2.23607 | 1.4577 | 1.0978 | 1.45766 | 1.09779 | 60 | 45.8258 | 166.67 | 11.547 |
| Native | Asteraceae | *Pulicaria_inuloides* | 2 | 24.3333 | 7.50555 | 1.5578 | 0.1633 | 1.55779 | 0.16331 | 71 | 5.65685 | 210 | 0 |
| Native | Asteraceae | *Pulicaria_undulata* | 15 | 39.8571 | 6.66905 | 1.2423 | 0.7619 | 1.24233 | 0.76187 | 42 | 53.6159 | 277.5 | 117.011 |
| Native | Tamaricaceae | *Reaumuria_alternifolia* | 1 | 16 | 1.73205 | 2.1964 | 0.0294 | 2.19644 | 0.02941 | 115 | 162.635 | 186.5 | 37.4767 |
| Native | Resedaceae | *Reseda_muricata* | 9 | 17.1429 | 3.57904 | 1.6868 | 0.168 | 1.68676 | 0.16804 | 88.75 | 35.678 | 447.5 | 83.0161 |
| Native | Resedaceae | *Reseda_pruinosa* | 5 | 18 | 1.1547 | 1.9096 | 0.0455 | 1.90965 | 0.04545 | 139 | 85.6329 | 129.33 | 8.0829 |
| Native | Fabaceae | *Retama_raetam* | 10 | 160.667 | 42.923 | 1.6085 | 0.5445 | 1.60849 | 0.54449 | 64.2222 | 65.7605 | 775.56 | 125.908 |
| Native | Rosaceae | *Rosa_arabica* | 1 | 80 | 0 | 1.3036 | 0 | 1.3036 | 0 | 0 | NA | 120 | NA |
| Native | Lamiaceae | *Salvia_multicaulis* | 14 | 15.5 | 9.24276 | 1.7058 | 0.2496 | 1.70577 | 0.24959 | 14.75 | 29.5 | 43.25 | 25.1313 |
| Native | Asteraceae | *Seriphidium_herba.album* | 39 | 30.1111 | 15.8486 | 1.679 | 0.4818 | 1.67896 | 0.4818 | 97.0667 | 78.1724 | 533.8 | 372.293 |
| Native | Brassicaceae | *Sisymbrium_erysimoides* | 32 | 12.1818 | 4.70783 | 1.5641 | 0.4606 | 1.56411 | 0.46061 | 13.8333 | 24.2439 | 17.167 | 6.33772 |
| Native | Brassicaceae | *Sisymbrium_irio* | 6 | 18.3333 | 14.4338 | 1.6066 | 0.2132 | 1.6066 | 0.21317 | 69.5 | 91.2168 | 15 | 1.41421 |
| Native | Solanaceae | *Solanum_nigrum* | 15 | 41.1667 | 14.1089 | 1.4964 | 0.2667 | 1.49644 | 0.26673 | 84.1429 | 99.9974 | 75.429 | 30.0381 |
| Native | Solanaceae | *Solanum_sinaicum* | 3 | 58 | 2.3094 | 1.493 | 0.1805 | 1.49304 | 0.18054 | 27 | 4.24264 | 93 | 38.1838 |
| Native | Solanaceae | *Solanum_villosum* | 9 | 30.2 | 2.86356 | 1.0055 | 0.6679 | 1.00548 | 0.66786 | 63.6667 | 58.3809 | 111.67 | 11.5036 |
| Native | Asteraceae | *Sonchus_asper* | 3 | 7 | 0 | 0.9505 | 0.5496 | 0.95052 | 0.54961 | 5 | 8.66025 | 17.667 | 4.72582 |
| Native | Asteraceae | *Sonchus_oleraceus* | 35 | 8 | 4.20526 | 1.167 | 0.4999 | 1.16695 | 0.4999 | 53.1 | 85.0587 | 22 | 14.6667 |
| Native | Poaceae | *Sorghum_virgatum* | 12 | 78 | 5.7735 | 1.5093 | 0.2826 | 1.50927 | 0.28255 | 61 | 24.0416 | 27 | 9.89949 |
| Native | Lamiaceae | *Stachys_aegyptiaca* | 17 | 31.5 | 5.52914 | 1.4732 | 0.2354 | 1.47315 | 0.23539 | 50.4 | 29.8798 | 263.6 | 52.4099 |
| Native | Poaceae | *Stipa_arabica* | 3 | 23 | 0 | 1.7304 | 0 | 1.73041 | 0 | 0 | NA | 40 | NA |
| Native | Poaceae | *Stipa_capensis* | 10 | 23 | 0 | 1.2179 | 0 | 1.21793 | 0 | 90 | NA | 45 | NA |
| Native | Poaceae | *Stipa_parviflora* | 20 | 61.5 | 44.456 | 1.9131 | 0.6873 | 1.91314 | 0.68725 | 650 | 919.239 | 27 | 9.89949 |
| Native | Poaceae | *Stipagrostis_ciliata* | 8 | 12.6667 | 1.1547 | 1.9233 | 0.0949 | 1.9233 | 0.09489 | 177.5 | 30.4056 | 13 | 1.41421 |
| Native | Chenopodiaceae | *Suaeda_aegyptiaca* | 4 | 34 | 0 | 1.5245 | 0 | 1.52452 | 0 | 56 | NA | 180 | NA |
| Native | Poaceae | *Taeniatherum_caput.medusae* | 8 | 27.4 | 21.7784 | 1.4868 | 0.7688 | 1.48679 | 0.76884 | 103 | 106.417 | 127.5 | 220.343 |
| Native | Tamaricaceae | *Tamarix_aphylla* | 1 | 200 | 0 | 1.3649 | 0 | 1.36494 | 0 | 50 | NA | 580 | NA |
| Native | Tamaricaceae | *Tamarix_nilotica* | 4 | 199.167 | 25.3804 | 1.8468 | 0.1807 | 1.84677 | 0.18066 | 12 | 13.8564 | 769.25 | 162.549 |
| Native | Asteraceae | *Tanacetum_sinaicum* | 1 | 250 | 0 | 1.951 | 0 | 1.95095 | 0 | 45 | NA | 1105 | NA |
| Native | Lamiaceae | *Teucrium_polium* | 7 | 20.25 | 3.77492 | 1.7223 | 0.125 | 1.72235 | 0.12501 | 56.6667 | 86.3095 | 196 | 129.047 |
| Native | Zygophyllaceae | *Tribulus_terrestris* | 3 | 3 | 0 | 1.2981 | 0 | 1.29808 | 0 | 5 | NA | 88 | NA |
| Native | Boraginaceae | *Trichodesma_africanum* | 12 | 19 | 6.06218 | 1.4635 | 0.4696 | 1.46351 | 0.46957 | 46.8333 | 41.6817 | 150.33 | 37.8717 |
| Native | Scrophulariaceae | *Verbascum_sinaiticum* | 37 | 57.0588 | 70.8347 | 1.4901 | 0.5794 | 1.49006 | 0.57943 | 126.818 | 247.908 | 72.455 | 39.5028 |
| Native | Fabaceae | *Vicia_monantha* | 5 | 10 | 0 | 1.9918 | 0 | 1.9918 | 0 | 8 | NA | 28 | NA |
| Native | Solanaceae | *Withania_somnifera* | 6 | 86.9 | 28.6374 | 1.6816 | 0.2484 | 1.6816 | 0.24843 | 112.167 | 64.8981 | 656.67 | 119.108 |
| Native | Brassicaceae | *Zilla_spinosa* | 82 | 56.02 | 28.9105 | 1.9593 | 1.0417 | 1.82405 | 0.56646 | 139.2 | 275.42 | 228.57 | 104.237 |
| Native | Rhamnaceae | *Ziziphus_spina.christi* | 6 | 95.6 | 39.4045 | 1.4601 | 0.3654 | 1.46006 | 0.36535 | 28 | 38.3406 | 710 | 217.371 |
| Native | Zygophyllaceae | *Zygophyllum_coccineum* | 1 | 23 | 0 | 1.9037 | 0 | 1.90373 | 0 | 0 | NA | 345 | NA |

References

1. Ayyad, M. A., Fakhry, A. M. & Moustafa, A.-R. A. Plant biodiversity in the Saint Catherine area of the Sinai peninsula, Egypt. 17 (2000).

2. Basuki, T. M., Van Laake, P. E., Skidmore, A. K. & Hussin, Y. A. Allometric equations for estimating the above-ground biomass in tropical lowland Dipterocarp forests. *For. Ecol. Manage.* **257**, 1684–1694 (2009).
